# A randomized controlled trial of mindfulness: effects on academic stress, academic burnout, and psychological resilience in university students

**DOI:** 10.3389/fpsyg.2025.1722669

**Published:** 2025-11-27

**Authors:** Shanwei Chen, Xiuzhi Qi

**Affiliations:** 1 College of Education, Baoji University of Arts and Sciences, Baoji, China; 2 Academy of Fine Arts, Baoji University of Arts and Sciences, Baoji, China

**Keywords:** mindfulness training, academic stress, academic burnout, psychological resilience, randomized controlled trial, university students

## Abstract

**Introduction:**

Academic stress, academic burnout, and reduced psychological resilience are prevalent among university students, particularly during examination periods. This study evaluated the effectiveness of an 8-week structured mindfulness training program in improving these outcomes.

**Methods:**

A randomized controlled trial (RCT) was conducted with 153 undergraduate and graduate students who were randomly assigned to a mindfulness intervention group (*n* = 77) or a minimal-contact control group (*n* = 76). Intention-to-treat (ITT) analysis was performed using linear mixed models with likelihood-based estimation under the missing-at-random assumption.

**Results:**

At the primary endpoint (Week 8), the intervention group showed significantly lower academic stress compared with the control group (*B* = 14.79, 95% CI [12.96, 16.62], *p* < 0.001, Cohen's *d* = −1.89). Secondary outcomes demonstrated parallel benefits, including reduced academic burnout (*B* = 11.31, 95% CI [8.62, 14.00], *p* < 0.001, *d* = −1.26) and increased psychological resilience (*B* = −18.42, 95% CI [−20.94, −15.90], *p* < 0.001, *d* = 2.02). These effects were largely sustained at the 2-week follow-up.

**Discussion:**

The findings indicate that structured mindfulness training significantly decreases academic stress and burnout while enhancing psychological resilience among Chinese university students. These results support the value of integrating mindfulness-based programs into higher education mental health initiatives.

## 1 Introduction

In recent years, increasing attention has been paid to the mental health of university students, particularly during high-pressure academic contexts such as examination periods. During these times, academic stress tends to rise sharply, leading to psychological problems including anxiety, depression, and sleep disturbances (Dyrbye et al., 2006). Recent studies have further highlighted that academic stress is associated with physical manifestations such as chronic fatigue, sleep disruption, and immune suppression (Barbayannis et al., 2022; Córdova Olivera et al., 2023), and longitudinal research among Chinese students has revealed significant impacts of lifestyle factors on stress-related health outcomes. Moreover, kindness-based and mindfulness-related contemplative practices have been shown to promote psychological well-being and reduce stress-related symptoms across diverse populations (Galante et al., 2014).

Among the negative consequences of academic stress, academic burnout is particularly prominent. It is characterized by emotional exhaustion, reduced academic efficacy, and a negative or detached attitude toward learning, which severely undermines students' learning motivation and psychological wellbeing (Schaufeli et al., 2002). Specifically, emotional exhaustion refers to feelings of being emotionally drained and fatigued due to academic demands. Academic efficacy reflects students' perceived competence and sense of accomplishment in their studies, with lower efficacy indicating higher burnout. Cynicism represents a detached or negative attitude toward learning and academic activities. These three dimensions together constitute the core framework of academic burnout. In contrast, psychological resilience has been shown to play a protective role in buffering the adverse effects of academic stress and burnout (Connor and Davidson, 2003), enabling students to maintain psychological stability and positive adaptation in the face of adversity. Recent longitudinal evidence further indicates that resilience mediates the relationship between mindfulness, self-compassion, and psychological distress, underscoring its importance in stress-buffering processes (Ueno and Amemiya, 2024).

Recent meta-analytic evidence supports the role of resilience as a potential mechanism in coping with academic stress, highlighting its importance for intervention programs (Koppenborg et al., 2024; Mulati and Purwandari, 2022). Mindfulness training, which originated from Eastern contemplative traditions and has been integrated into Western psychology, focuses on cultivating awareness and acceptance of present-moment experiences. It has demonstrated significant effectiveness in reducing stress, anxiety, and depression (Baer, 2003; Kabat-Zinn and Hanh, 2009; Shapiro et al., 2007). Meta-analyses focused on student populations have shown that Mindfulness-Based Interventions (MBIs) effectively reduce perceived stress and burnout while enhancing resilience and wellbeing (Dawson et al., 2020). Recent findings also highlight resilience as a key pathway through which mindfulness contributes to improved mental health outcomes (Sinha, 2025).

Nevertheless, systematic empirical research examining the comprehensive effects of mindfulness training on academic stress, academic burnout, and psychological resilience during examination periods among Chinese university students remains limited, and prior studies are primarily small-scale pilot investigations (Cheng and Lin, 2023; Rusadi et al., 2023). Although existing studies have examined the effects of mindfulness training on emotional symptoms, relatively few have integrated academic stress, academic burnout, and psychological resilience into a unified framework. Such integrative research can provide a more comprehensive understanding of the mechanisms underlying mindfulness interventions and inform evidence-based strategies for mental health promotion in higher education.

In the context of mainland China's highly competitive examination system, students may experience intervention effects distinct from those observed in Western educational settings (Han et al., 2024). This underscores the theoretical and practical value of exploring mindfulness interventions within Chinese cultural and academic environments.

To address these gaps, the present Randomized Controlled Trial (RCT) investigated the effects of an 8 week structured mindfulness training program on university students during examination periods. The primary outcome was academic stress, while secondary outcomes included academic burnout and psychological resilience.

Based on previous evidence, we proposed the following hypotheses:

(1) H1: Mindfulness training will significantly reduce academic stress compared with the control group.

(2) H2: Mindfulness training will significantly reduce academic burnout, including emotional exhaustion, academic efficacy, and cynicism dimensions.

(3) H3: Mindfulness training will significantly enhance psychological resilience compared with the control group. Although no formal mediation was conducted, resilience may serve as a potential mechanism linking mindfulness training to reduced academic stress.

## 2 Methodology

### 2.1 Design and participants

This study adopted an RCT design. Participants were under graduate and graduate students enrolled in the School of Education at Baoji University of Arts and Sciences. Recruitment was conducted through the university's Mental Health Center, class WeChat groups, and campus posters. Eligible students were invited to participate voluntarily. A total of 153 students meeting the inclusion criteria were recruited and randomly assigned to either the mindfulness experimental group (*n* = 77) or the control group (*n* = 76) using a computer-generated randomization procedure, with an allocation ratio of approximately 1:1.

Randomization was conducted by an independent researcher who was not involved in participant recruitment or outcome assessment, and allocation concealment was maintained using sequentially numbered, opaque, sealed envelopes. Sequentially numbered, opaque, sealed envelopes were used to assign participants to groups. Outcome assessors were blinded to group allocation, although participant blinding was not feasible due to the behavioral nature of the intervention.

During the study, 82 participants (53.6%) completed the post-intervention assessment at Week 8 (T8) and were included in the final analyses, including 43 in the experimental group and 39 in the control group. A total of 71 participants (46.4%) dropped out before completing the post-intervention assessment. The primary reasons for attrition included lack of time due to academic workload and voluntary withdrawal without stated reasons.

Intervention fidelity and participant attrition were systematically monitored throughout the 8 week program. As shown in Table 1, completers in the experimental group (*n* = 43) attended all 8 scheduled sessions (100% attendance rate; mean duration = 90 min per session), while dropouts (n = 34) attended an average of 3.6 sessions (range: 1–5; 45.2% attendance rate). Session duration was consistently maintained at the preset 90 mins.

**Table 1:** RCT intervention fidelity core statistics.

**Statistical Items**	**EG (Completer)**	**EG (Dropout)**
Sample size (*n*)	43	34
Preset total sessions	8	8
Mean actual attended sessions	8.0	3.6
Attended sessions (min-max)	8-8	1-5
Attendance rate (%)	100	45.22
Mean session duration (min)	90	90
Preset session duration (min)	90	90
Remarks	All participants completed all 8 sessions	Participants dropped out gradually; with 22 reporting a lack of time and 12 reporting voluntary withdrawal.

EG, Experimental group.

As shown in Table 2, participant dropout occurred progressively during the 8 week intervention, reaching 46.4% by Week 8. The main reasons for attrition were lack of time due to academic workload (63.4%) and voluntary withdrawal without specific reasons (36.6%). Attrition rates were comparable between groups, with Mindfulness at 44.2% and Control at 48.7%, and the distribution of dropout reasons did not differ significantly (*p* = 1.00, ϕ = 0.00, Δ% = 2.5%). Furthermore, baseline comparisons between completers and dropouts revealed no significant differences in demographic or psychological variables, suggesting that attrition was largely random and unlikely to bias the intervention results.

**Table 2:** Distribution of dropout reasons by group.

**Reason**	**EG (*n* = 34)**	**CG (*n* = 37)**	**Total (*n* = 71)**	***p***	**ϕ**	**Δ%**
**Lack of time**	22 (64.7%)	23 (62.2%)	45 (63.4%)	1.00	0.00	2.5%
**Voluntary withdrawal**	12 (35.3%)	14 (37.8%)	26 (36.6%)	-	-	-
**Total**	34	37	71	-	-	-

EG, Experimental group; CG, Control group.

To adhere to the Intention-To-Treat (ITT) principle, all 153 randomized participants were included in the primary analysis using Linear Mixed Model (LMM) and likelihood-based estimation to handle missing data. Completers-only analyses were conducted as sensitivity checks.

Ethical approval for this study was obtained from the Human Research Ethics Committee of the School of Education, Baoji University of Arts and Sciences (Approval No. BJWLXY-EDU-2024-012), granted on April 12, 2024, and valid until July 1, 2024. The study procedures adhered strictly to ethical guidelines, ensuring full protection of participants' rights and confidentiality.

The inclusion criteria included: participants had to be at least 18 years old; must be currently preparing for final or qualification examinations; and self-reported absence of any severe psychiatric disorders.

The exclusion criteria included: participants who had taken part in a structured mindfulness program within the previous 3 months; individuals with a history of severe depression, anxiety, or psychotic disorders; individuals currently receiving psychotherapy or pharmacological treatment for psychiatric disorders; and inability to commit to attending all sessions during the 8 week intervention period.

The initial sample-size estimation was conducted using *G*Power 3.1 based on an independent-samples *t*-test (two-tailed, *d* = 0.5, α = 0.05, power = 0.80), indicating a minimum of 40 participants per group. At the early design stage, this calculation was adopted as a conservative approximation because the longitudinal LMM had not yet been finalized as the primary analytical approach. To account for possible attrition, 153 participants were randomized to ensure sufficient power. However, an attrition rate of 46% during the intervention resulted in a reduction of the number of completers to 82. Although a formal simulation-based power analysis for the LMM was not performed, a *post-hoc* evaluation suggested that the achieved sample still provided moderate statistical power for detecting medium-sized group × time interaction effects across repeated measures, consistent with prior mindfulness intervention research (Wagner et al., 2023). The discrepancy between the planned enrollment of approximately 100 and the actual enrollment of 153 resulted from unexpectedly high student interest and an intentional oversampling strategy aimed at mitigating anticipated dropout.

To ensure the adequacy of the sample size for the final analytical framework, a *post-hoc* statistical power evaluation was conducted to approximate the achieved sensitivity under the LMM design. Based on recognized approximations (Liu and Liang, 1997), a two-group repeated-measures LMM with three major measurement points (T0, T8, and Follow-up), assuming an intraclass correlation (ρ) of 0.40 and a medium effect size (f = 0.25), yields an estimated achieved power of approximately 0.78 with *n* = 82 completers and >0.85 under the full ITT dataset (*n* = 153). These *post-hoc* results indicate that the study retained adequate power for detecting medium-sized group × time interaction effects, despite attrition.

Although the dropout rate (46.4%) inevitably reduced the effective sample size and slightly widened confidence intervals, attrition was balanced across groups (Δ% = 2.5%), and baseline comparisons between completers and dropouts showed no significant differences in demographic or psychological variables (all *p* >0.10), suggesting that missingness was largely random and unlikely to bias group-level estimates. Nonetheless, this limitation was explicitly considered in interpreting effect sizes and generalizability, as discussed in the Limitations section.

The control group received weekly “mental health information” as a minimal active control condition, consisting of 15–20 min of written materials and short videos on general psychological wellbeing, delivered via WeChat each week. The content aimed to provide psychoeducation without specific mindfulness strategies. Potential expectancy effects and minor contamination due to exposure to mental health information are acknowledged and discussed in the limitations section.

For randomization, a computer-generated random number table was used to allocate participants. All eligible participants were assigned an ID number and randomly allocated to either the experimental or control group. The randomization process ensured that the two groups were balanced on key demographic variables, including gender, age, and academic year.

**Figure 1:**
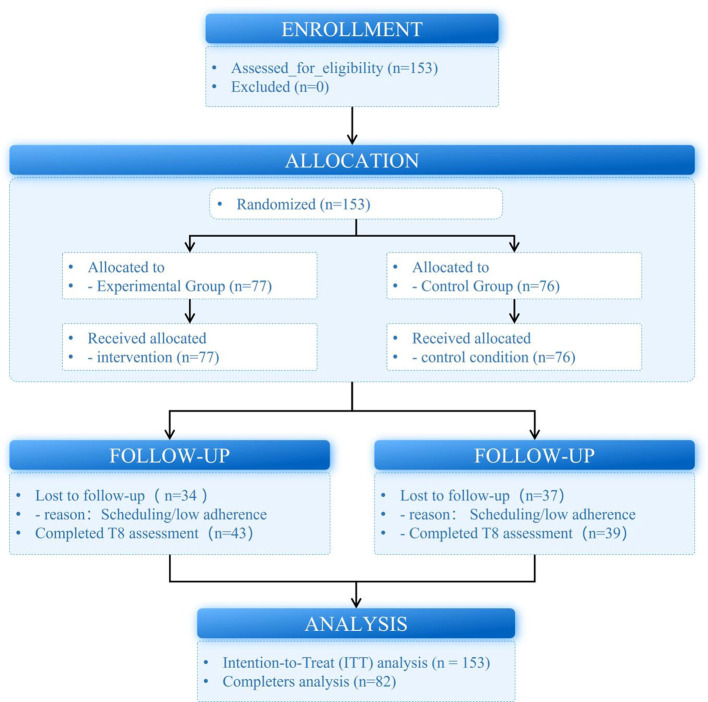
CONSORT Diagram of Participant Progression.

Figure 1. was used to summarize participant enrollment, allocation, follow-up, and analysis, including reasons for dropout at each stage and by group.

Table 3 presents the baseline characteristics of all randomized participants (*n* = 153), including gender, age group, education level, and self-reported anxiety and depression. Overall, 56.2% of the sample were female and 43.8% were male. Most participants were between 18 and 24 years old (62.1%), followed by 25–30 years old (27.5%) and over 30 years old (10.5%). In terms of education level, 64.1% were undergraduates and 35.9% were postgraduates. The proportion of participants reporting anxiety or depression was relatively low: 9.8% reported anxiety, 3.3% reported depression, 5.9% reported both, and 48.4% did not report either condition.

**Table 3:** Baseline characteristics of all randomized participants.

**Variable**	**Category**	**Total**		**EG**		**CG**	
		***n***	**%**	***n***	**%**	***n***	**%**
Sex (*n* = 153)	Female	89	58.2	44	57.1	45	59.2
	Male	64	41.8	33	42.9	31	40.8
Age group (*n* = 153)	18–24 years old	73	47.7	41	53.2	32	42.1
	25–30 years old	61	39.9	28	36.4	33	43.4
	Over 30 years old	19	12.4	8	10.4	11	14.5
Education (*n* = 153)	Undergraduate student	98	64.1	52	67.5	46	60.5
	Graduate student	55	35.9	25	32.5	30	39.5
Self-reported disorder	Anxiety	15	9.8	9	11.7	6	7.9
	Depression	5	3.3	3	3.90	2	2.6
	Anxiety and Depression	9	5.9	4	5.20	5	6.6
	Does not present	50	32.7	25	33.8	24	31.6
	Did not declare	74	48.4	35	45.5	39	51.3

EG, Experimental group; CG, Control group; M, Mean; SD, Standard deviation.

### 2.2 Experimental procedure

The experimental procedure consisted of four phases: Pre-test, Intervention, *Post-test*, and a 2 week Follow-up, lasting a total of 11 weeks (see Figure 1 for the experimental flowchart).

In the pre-test phase (Week 0), both the Experimental and control groups completed baseline assessments, which included a demographic questionnaire, the Academic Burnout Scale, the Academic Stress Scale, and the Psychological Resilience Scale. Measurements were conducted in quiet classroom settings using either paper-based or online questionnaires, ensuring independence and anonymity in responses.

The intervention phase lasted for 8 weeks (Weeks 1–8). The Experimental group received a structured mindfulness training program consisting of weekly 90 min group sessions and daily 15-min individual practices. Participants were required to check in and provide feedback on their daily practice through a WeChat group. The control group maintained their usual study and daily routines without receiving mindfulness training; however, the research team sent weekly mental health information to maintain engagement and reduce attrition.

The *post-test* phase took place at the end of Week 8, using the same set of scales as in the pre-test to assess the effects of the intervention. All data were independently entered and verified by two research assistants who were not involved in the intervention to ensure data accuracy and integrity.

2 weeks after the intervention, participants were assessed again using the same questionnaires to examine the sustainability and possible delayed effects of the mindfulness training.

### 2.3 Measurement instruments and indicators

Multiple scales were employed in this study to assess participants' demographic information, academic burnout, academic stress, and psychological resilience. All instruments used were Chinese versions that had undergone translation, back-translation, expert review, and pilot testing to ensure cultural equivalence, validity, and reliability.

Questionnaires were completed within a standardized time frame, either in paper-based or online format, under the on-site guidance and supervision of research assistants to ensure data quality. Data entry was performed independently by two individuals and cross-checked. Missing values were handled using item mean substitution, and only questionnaires with less than 20% missing data were retained for analysis. Cronbach's α coefficients were calculated for each scale to assess internal consistency and ensure measurement reliability.

#### 2.3.1 Demographic questionnaire

The demographic questionnaire collected basic participant information, including gender, age, grade level, major, exam preparation status, mental health history, and experience with mindfulness training. All variables were uniformly coded for subsequent statistical analysis. Participants provided informed consent before completing the questionnaire, which took approximately 5–8 mins. The collected data were mainly used to describe the characteristics of the sample and to serve as covariates in later analyses.

The main content and example coding are as follows: ID (participant number); Gender (0 = female, 1 = male, 2 = prefer not to disclose); Age (integer, in years); Grade (Undergraduate Year 1, Year 2, Year 3, Year 4, or Graduate Year 1, Year 2); Major (one of four undergraduate majors within the School of Education; graduate students provided their specific major); Currently preparing for exams (1 = yes, 0 = no); History of mental disorders (1 = yes, 0 = no; if yes, specify whether currently receiving medication or psychotherapy); Previous structured mindfulness training (1 = yes, 0 = no); Major life events in the past 3 months (1 = yes, 0 = no; if yes, briefly describe the event).

#### 2.3.2 Academic burnout scale

Academic burnout was assessed using the Maslach Burnout Inventory–Student Survey (MBI-SS), which includes three dimensions: Emotional Exhaustion (EE), Cynicism (CY), and Academic Efficacy (AE). Numerous cross-national studies have demonstrated that the MBI-SS has good reliability and structural validity among student populations (Portoghese et al., 2018).

The scale consists of 15 items, rated on a 7-point frequency scale ranging from 0 (never) to 6 (always). The EE subscale contains 5 items, reflecting the degree of emotional fatigue resulting from academic demands; The CY subscale includes 4 items, measuring detachment and negative attitudes toward learning; The AE subscale comprises 6 items, assessing positive academic achievement and self-efficacy.

In this study, AE is a positively oriented dimension, where higher scores indicate greater efficacy and lower burnout. In contrast, higher scores on EE and CY indicate higher burnout. For dimensional analyses, AE was analyzed in its original orientation to reflect adaptive functioning, whereas the total Academic Burnout score, which was computed as the sum of all 15 items, was interpreted in the direction of overall burnout, which means higher values indicate greater burnout. This scoring structure was consistently applied across analyses, tables, and figures to maintain interpretive clarity.

The Chinese version of the MBI-SS was translated and back-translated by bilingual experts, followed by a pilot test among Chinese university students to ensure cultural and linguistic equivalence. Its reliability and structural validity were verified using Cronbach's α coefficients across all measurement occasions. Specifically, α for the total scale were: T0 = 0.826, T1 = 0.814, T2 = 0.832, T3 = 0.857, T4 = 0.851, T5 = 0.860, T6 = 0.860, T7 = 0.823, T8 = 0.799, and Follow-up = 0.723. These coefficients indicate satisfactory to good internal consistency at most time points, with minor reductions at T8 and Follow-up.

McDonald's ω was not calculated, as Cronbach's α remains the most widely reported reliability index for the MBI-SS in prior studies and provides a sufficient estimate of internal consistency for the present analyses. Multilevel reliability indices were beyond the scope of this study but are recommended for future longitudinal investigations.

#### 2.3.3 Academic stress scale

Academic stress was assessed using the Academic Stress Scale (ASS). The original scale contains 35 items rated on a 5-point Likert scale (1–5), with higher scores indicating greater perceived academic stress (Kohn and Frazer, 1986). The Chinese version was adapted and localized based on the original instrument, ultimately retaining 15 items to better fit the academic context of Chinese universities. Previous research has supported the reliability and structural validity of related psychological measures in Chinese college students, providing a methodological basis for the use of the adapted ASS in this population (Lu et al., 2018).

Participants completed the questionnaire based on their academic experiences during the past week. Missing data were handled using item-mean substitution. The scale can be used to assess overall academic stress through the total score, or to analyze specific subdomains such as “Academic Stress,” “Assignment and Time Management Stress,” “Teacher–Student Relationship Stress,” and “Academic Expectation Stress,” which are useful for subsequent factor analyses and targeted interventions. Each item is rated from 1 (no stress or not at all distressing) to 5 (extremely stressful or highly distressing), resulting in a total score range of 15–75. A consistent temporal reference was applied during data processing to ensure comparability across participants. Internal consistency of the total scale in the current sample was: T0 = 0.781, T1 = 0.846, T2 = 0.837, T3 = 0.828, T4 = 0.832, T5 = 0.824, T6 = 0.796, T7 = 0.800, T8 = 0.774, Follow-up = 0.778. These α values indicate good reliability across the study period.

However, because this 15-item version was adapted from the original 35-item Academic Stress Scale, its structural validity and longitudinal measurement invariance were not formally examined in the present study. Future research should conduct confirmatory factor analysis and invariance testing to verify factorial stability across time and groups.

#### 2.3.4 Psychological resilience scale

Psychological resilience was measured using the Chinese version of the Connor–Davidson Resilience Scale (CD-RISC). This scale includes 25 items, rated on a 5-point Likert scale from 0 (“not true at all”) to 4 (“true nearly all the time”), with higher scores reflecting greater psychological resilience (Yu and Zhang, 2007). The Chinese CD-RISC has been repeatedly validated among university students in China, demonstrating good reliability and structural validity (Zhang et al., 2018).

The total score ranges from 0 to 100, and can be further divided into sub dimensions such as “Tenacity,” “Strength,” and “Optimism,” depending on research needs. Scores can be analyzed both as an overall indicator of resilience and as a positive psychological trait in mediation or moderation analyses. Participants completed the scale based on their actual experiences over the past week. Data quality control and reliability testing followed the same procedures as for the previous scales. α coefficients for the total scale were: T0 = 0.851, T1 = 0.838, T2 = 0.839, T3 = 0.836, T4 = 0.865, T5 = 0.836, T6 = 0.826, T7 = 0.812, T8 = 0.817, Follow-up = 0.800; McDonald's ω was not calculated for the same reasons described in Section 2.3.2. These α values demonstrate excellent internal consistency across all measurement points.

### 2.4 Mindfulness training intervention program

The study implemented an 8 week structured mindfulness training program that combined theoretical instruction with practical exercises, aiming to help university students alleviate academic stress and enhance psychological resilience. The intervention was based on the classical frameworks of Mindfulness-Based Stress Reduction (MBSR) and Mindfulness-Based Relapse Prevention (MBRP), and was adapted with reference to the intervention parameters and operational guidelines proposed by (Crane et al., 2017) to better suit the practical needs and schedules of college students. Specific adaptations included limiting each session to 90 mins, slightly shorter than the original protocols, with an emphasis on mindfulness practices targeting students' stress responses and coping strategies.

During the intervention period, one group training session per week was conducted, integrating theoretical lectures, mindfulness exercises, and group sharing. Sessions were scheduled during students' spare time to avoid conflicts with formal coursework, ensuring high participation rates.

Each session consisted of three parts, The first part, lasting approximately 30 mins, focused on theoretical instruction, introducing the fundamental concepts and core principles of mindfulness, its relationship with stress and emotion regulation, and incorporating case discussions to help students understand the scientific basis and applied value of mindfulness; The second part, lasting about 40 mins, involved mindfulness practice, including guided exercises such as breath awareness, body scan, and emotion observation, as well as dynamic mindfulness practices like walking meditation, with the exercises progressing gradually from basic to advanced levels. The final part, approximately 20 mins, consisted of group sharing and feedback, where participants were encouraged to discuss their practice experiences, challenges, and insights, fostering self-reflection and social support within the group.

The weekly course themes were structured as follows: In Week 1, participants were introduced to mindfulness and breath awareness; Week 2 focused on body scanning and identifying sources of stress; Week 3 emphasized emotion awareness and acceptance; Week 4 introduced mindfulness strategies for coping with stress; In Week 5, participants practiced self-compassion and cultivating positive emotions; Week 6 concentrated on observing thoughts and cognitive defusion; Week 7 explored the integration of mindfulness with action; Week 8 concluded the program with a review and future planning, reflecting on practice achievements and formulating a personal practice plan and coping strategies.

Sessions were led by instructors with formal professional training in mindfulness and a background in counseling or education. Each instructor has received at least 40 h of structured MBSR/MBRP training. Instructors held relevant certifications and received ongoing supervision throughout the intervention. Participant adherence was tracked through daily practice logs and attendance records, with a mean of 63.26 mins per day and an average of 70.3% session attendance; participants attending ≥75% of sessions were considered per-protocol (PP). No adverse events were reported during the intervention period. Participants in the control group received weekly mental health information via WeChat, consisting of psychoeducational materials and short videos on general mental wellbeing. This was designed as a minimal active control condition to balance attention and engagement while avoiding mindfulness-specific components. The frequency and delivery were standardized, and participants were instructed not to engage in mindfulness activities during the 8 week period.

Figure 1 illustrates the CONSORT diagram of participant progression through the study, from recruitment and baseline assessment (T0) to randomization, the 8 week intervention, post-intervention assessment (T8), and inclusion in the analyses. A total of 153 eligible participants were randomly assigned to the experimental group (*n* = 77) or control group (*n* = 76). The experimental group received weekly 90 min mindfulness sessions, while the control group maintained their regular academic schedule. At T8, 43 participants in the experimental group, of which 34 were lost to follow-up, 33 due to scheduling conflicts or low adherence, and 39 in the control group, of which 37 were lost to follow-up, 38 due to scheduling conflicts or low adherence, completed the assessments.

Participant attrition occurred progressively during the 8 week intervention, resulting in 82 participants (53.6%) completing the post-intervention assessment at Week 8 (T8). The primary ITT analysis included all 153 randomized participants using LMM with likelihood-based estimation to handle missing data, while completers-only analyses (*n* = 82) were conducted as sensitivity checks. Figure 1 shows participant progression and reasons for dropout by group.

### 2.5 Data analysis

The data analysis for this study was conducted in three stages: data preprocessing, descriptive statistics, and inferential statistical analysis. Primary analyses adhered to the ITT principle, including all 153 randomized participants, while completers-only data (*n* = 82) were used for sensitivity analyses to examine robustness. Given an overall dropout rate of approximately 46%, a coherent missing-data strategy combining multiple imputation (MI) and likelihood-based estimation under the Missing at Random (MAR) assumption was adopted to ensure valid ITT estimation despite attrition.

Specifically, item-level missingness below 5% was imputed using the Expectation-Maximization (EM) algorithm, whereas higher levels of scale-level missingness were multiply imputed (*m* = 5) using predictive mean matching before the ITT analyses. Longitudinal missingness was handled directly by LMM through likelihood-based estimation, which retains all available observations without listwise deletion. All analyses were performed using SPSS 26.0 and Python 3.10.

#### 2.5.1 Data preprocessing

All collected questionnaire data were carefully inspected for completeness and consistency. Responses with logical errors or extreme inconsistencies were excluded after double-checking raw data logs. Outliers were identified using boxplots and Z-scores (|Z| > 3.29). To handle missing data, a two-step imputation strategy was applied consistently across all analyses.

At the item level, missingness below 5% was estimated using the EM algorithm; when the proportion of missing items within a scale exceeded 5%, multiple imputation (m = 5) via predictive mean matching was applied. The imputed datasets were then analyzed using LMM with Restricted Maximum Likelihood (REML) estimation under the MAR assumption, which properly accounts for time-level missingness. Model diagnostics confirmed that imputation distributions closely matched observed data and that no systematic bias was introduced by the imputation procedure. All scales demonstrated acceptable to excellent internal consistency.

#### 2.5.2 Descriptive statistics

Baseline demographic and psychological characteristics were compared between the experimental and control groups. Chi-square tests were used for categorical variables, and independent-samples *t*-tests with Levene's correction were conducted for continuous variables. A full-sample (ITT) baseline comparison was conducted first to confirm successful randomization, followed by a completer–dropout comparison to check attrition bias.

To ensure transparency and numerical consistency, all descriptive statistics (means and standard deviations) were recalculated and presented by group and by time point in the main or supplementary tables, with corrected rounding and clear reporting of missing values. Descriptive statistics are summarized in Tables 4–6.

**Table 4:** Baseline Characteristics Between Experimental and Control Groups of Completer (*n* = 82).

**Variable**	**EG *(n* = 43)**	**%**	**CG (*n* = 39)**	**%**	**Test statistic**	***p***
Male	21	48.8	16	41.0	χ^2^ = 0.24	0.63
Female	22	51.2	23	59.0		
Age	23.1 ± 2.41	–	23.8 ± 2.44	–	*t* = −1.30	0.20
Age group:18–21	13	30.2	8	20.5	χ^2^ = 1.24	0.54
Age group:22–25	22	51.2	21	53.8		
Age group:26–30	8	18.6	10	25.6		

EG, Experimental group; CG, Control group.

**Table 5:** Baseline Comparisons of Psychological Constructs Between Experimental and Control Groups of Completer (*n* = 82).

**PC**	**EG (M)**	**EG (SD)**	**CG (M)**	**CG (SD)**	***t***	***df***	***p***	**Cohen's *d***
**Stress**	38.58	6.02	38.21	6.89	0.26	75.9	0.794	0.06
**Burnout**	40.14	10.17	41.62	9.94	−0.66	79.5	0.508	−0.15
**Resilience**	45.65	6.94	46.74	7.87	−0.66	76.2	0.509	−0.15

PC, Psychological constructs; EG, Experimental group; CG, Control group; M = Mean; SD = Standard deviation.

**Table 6:** Baseline comparability of all randomized participants.

**Variable**	**Category**	**EG Mean ±SD or %**	**CG Mean ±SD or %**	**Test Statistic**	***df***	***p***	**Cohen's *d***
Age (*n* = 153)		24.53 ± 4.70	25.32 ± 4.42	*t* = −1.06	151	0.290	−0.17
Stress_T0		38.14 ± 6.23	37.46 ± 7.30	*t* = 0.62	151	0.535	0.10
Burnout_T0		39.17 ± 10.65	40.92 ± 10.49	*t* = −1.03	151	0.307	−0.17
Resilience_T0		44.74 ± 7.42	46.00 ± 8.60	*t* = −0.97	151	0.334	−0.16
Sex (*n* = 153)	Female	57.1%	59.2%	χ^2^ = 0.009	1	0.924	-
	Male	42.9%	40.8%	χ^2^ = 0.009	1	0.924	-
Education (*n* = 153)	Graduate	32.5%	39.5%	χ^2^ = 0.539	1	0.463	-
	Undergraduate	67.5%	60.5%	χ^2^ = 0.539	1	0.463	-
Self-reported disorder	Anxiety	11.7%	7.9%	χ^2^ = 1.201	4	0.878	-
	Anxiety and Depression	5.2%	6.6%	-	-	-	-
	Depression	3.9%	2.6%	-	-	-	-
	Did not declare	45.5%	51.3%	-	-	-	-
	Does not present	33.8%	31.6%	-	-	-	-

#### 2.5.3 Inferential statistical analysis

To assess changes over time and between groups, LMM was fitted separately for each primary outcome (academic stress, burnout, and resilience) using the ITT dataset (n = 153).

Each model included fixed effects for Group (Experimental vs. Control), Time (T0–T8), and their interaction (Group × Time), as well as random intercepts for participants. Random slopes for Time were added when they significantly improved model fit, as assessed by the Akaike Information Criterion (AIC). The models were estimated using Restricted REML, and the Kenward–Roger correction was applied to adjust degrees of freedom for small-sample bias. An AR(1) was used to model temporal autocorrelation in repeated measures. Covariates were included as fixed effects to control demographic variance.

*Post-hoc* pairwise comparisons were performed at the prespecified endpoint (Week 8) using Estimated Marginal Means (EMM) to obtain adjusted between-group differences with 95% confidence intervals and standardized effect sizes. All completer-only models were re-estimated as sensitivity analyses using the same model structure to test the robustness of the main ITT results. False Discovery Rate (FDR) correction (Benjamini–Hochberg) was applied to control for multiple testing. All tests were two-tailed with a significance threshold of *p* < 0.05.

## 3 Experimental results

### 3.1 Descriptive analysis of baseline characteristics

Based on the participants who completed the post-intervention assessment (T8), a total of N = 82 valid participants were included in the final analysis, with n = 43 in the intervention group and n = 39 in the control group. There were no significant differences between the two groups in terms of baseline characteristics such as gender, age, and age group (gender: χ^2^ = 0.24, *p* = 0.63; age: *t* = −1.30, *p* = 0.20; age group: χ^2^ = 1.24, *p* = 0.54), indicating good baseline comparability between groups following random assignment. Additionally, independent-samples *t*-tests confirmed no significant differences in the primary psychological constructs at baseline, including academic stress (*t* = 0.26, *df* = 75.9, *p* = 0.794, Cohen's *d* = 0.06), academic burnout (t = −0.66, *df* = 79.5, *p* = 0.508, Cohen's *d* = −0.15), and psychological resilience (*t* = −0.66, *df* = 76.2, *p* = 0.509, Cohen's *d* = −0.15), further supporting the comparability of the intervention and control groups (see Table 4). The means, standard deviations, and age distributions of baseline psychological measures showed that both groups had similar values, with moderate variability and no extreme outliers. Detailed data are presented in Table 4, which summarizes the baseline characteristics of participants in the intervention and control groups. Table 5, which presents the baseline comparisons of psychological constructs.

To ensure the appropriateness of parametric tests reported in Table 5, we conducted Shapiro–Wilk tests. In the Mindfulness group, Academic Stress (*W* = 0.960, *p* = 0.141), Academic Burnout (*W* = 0.949, *p* = 0.056), and Psychological Resilience (*W* = 0.962, *p* = 0.169) did not significantly deviate from normality. In the Control group, Academic Burnout (*W* = 0.947, *p* = 0.064) and Psychological Resilience (*W* = 0.972, *p* = 0.433) were approximately normally distributed, whereas Academic Stress (*W* = 0.936, *p* = 0.028) showed a slight deviation. Overall, the baseline distributions of psychological measures were acceptable for parametric analyses. Independent-samples *t* revealed no significant differences between groups, supporting the comparability of the experimental and control groups before the intervention.

Table 6 presents the baseline characteristics of all randomized participants (*n* = 153), including age, sex, education, self-reported psychological disorders, and baseline scores for academic stress, burnout, and resilience. Statistical tests show no significant differences between the experimental and control groups, indicating successful randomization and baseline comparability.

As shown in Table 7, based on the full sample, baseline comparisons between the Mindfulness and Control groups showed no significant differences in age, gender, or psychological measures. Similarly, as shown in Table 8, comparisons between completers and dropouts revealed no significant baseline differences in these variables. These results indicate good overall comparability and minimal dropout bias in the study sample.

**Table 7:** Experimental vs. control baseline comparison of all randomized participants.

**Variable**	**EG Mean ±SD or %**	**CG Mean ±SD or %**	**Test statistic**	***df***	***p***	**Cohen's *d***
Age	24.53 ± 4.70	25.32 ± 4.42	*t* = −1.06	151	0.290	−0.17
Stress_T0	38.14 ± 6.23	37.46 ± 7.30	*t* = 0.62	151	0.535	0.10
Burnout_T0	39.17 ± 10.65	40.92 ± 10.49	*t* = −1.03	151	0.307	−0.17
Resilience_T0	44.74 ± 7.42	46.00 ± 8.60	*t* = −0.97	151	0.334	−0.16
Sex (M%)	42.9%	40.8%	χ^2^ = 0.009	1	0.924	-

**Table 8:** Completers vs Dropouts Baseline Comparison of All Randomized Participants.

**Variable**	**Completers Mean ±SD or %**	**Dropouts Mean ±SD or %**	**Test statistic**	***df***	***p***	**Cohen's *d***
Age	24.84 ± 4.78	24.99 ± 4.40	*t = −0.19*	151	0.846	−0.03
Stress_T0	38.40 ± 6.41	37.11 ± 7.14	*t* = 1.17	151	0.245	0.19
Burnout_T0	40.84 ± 10.03	39.11 ± 11.17	*t* = 1	151	0.319	0.16
Resilience_T0	46.17 ± 7.37	44.44 ± 8.68	*t* = 1.32	151	0.189	0.22
Sex (M%)	40.2%	43.7%	χ^2^ = 0.07	1	0.792	-

### 3.2 Intervention effects analysis

Primary outcome analyses were conducted under the ITT principle using the full randomized sample (*N* = 153), with missing data handled via MI preprocessing and likelihood-based estimation under MAR. To examine the weekly changes in academic burnout, psychological resilience, and academic stress, LMM was applied to all randomized participants, including both experimental and control groups.

The results indicated a progressive decrease in academic burnout and academic stress, as well as a steady improvement in psychological resilience, across the 8 week intervention period.

Compared to baseline (T0), significant within-group changes emerged from Week 2 onward and remained consistent at Week 8 and during follow-up. Psychological resilience was associated with a significant upward trend starting from Week 2, reaching its peak in Week 8, and remaining above baseline at follow-up. Similarly, perceived academic stress showed a declining trajectory, with statistically significant changes evident from Week 2 onward.

Between-group comparisons are provided in Table 9, which reports EMM, Group × Time coefficients, 95% confidence intervals, *p*, change from baseline, and standardized effect sizes for both Experimental and Control groups. The follow-up row indicates missing direct measurements with the line. These results indicate that the mindfulness condition was associated with larger and more consistent improvements than the control condition, with differences that were sustained at follow-up.

**Table 9:** Intention-to-Treat analysis: estimated marginal means and between-group differences at key time points (LMM, *n* = 153).

**Outcome**	**Week**	**EG M ±SE**	**CG M ±SE**	**Group × Time B**	**95% CI**	***p***	**Δ vs. T0 (EG)**	**Δ vs. T0 (CG)**	**Cohen's d**
Burnout	T0	39.19	40.89	9.5	[5.36, 13.64]	<0.001^***^	0	0	−0.19
	T1	38.28	41.37	8.11	[3.97, 12.25]	<0.001^***^	−0.91	0.49	−0.35
	T2	34.69	39.38	6.51	[2.23, 10.79]	0.003^**^	−4.5	−1.5	−0.52
	T3	35.24	39.73	6.71	[2.39, 11.02]	0.002^**^	−3.95	−1.15	−0.5
	T4	34.3	40.26	5.24	[0.75, 9.74]	0.022^*^	−4.89	−0.63	−0.66
	T5	33.25	39.5	4.95	[0.33, 9.57]	0.036^*^	−5.94	−1.38	−0.7
	T6	31.61	38.86	3.95	[-0.74, 8.64]	0.099	−7.58	−2.02	−0.81
	T7	27.65	38.09	0.76	[-3.97, 5.48]	0.754	−11.54	−2.79	−1.17
	T8	27.35	38.66	11.31	[8.62, 14.0]	<0.001^***^	−11.84	−2.23	−1.26
	Follow-up	27.77	38.97	-	-	-	−11.42	−1.92	−1.25
Stress	T0	38.21	37.6	15.09	[12.25, 17.94]	<0.001^***^	0	0	0.08
	T1	36.43	38.87	12.05	[9.21, 14.9]	<0.001^***^	−1.78	1.26	−0.31
	T2	35.2	37.66	12.03	[9.09, 14.98]	<0.001^***^	−3.01	0.05	−0.31
	T3	31.68	37.5	8.67	[5.71, 11.64]	<0.001^***^	−6.53	−0.11	−0.74
	T4	30.68	37.56	7.62	[4.52, 10.71]	<0.001^***^	−7.52	−0.05	−0.88
	T5	28.01	37.64	4.87	[1.69, 8.04]	0.003^**^	−10.19	0.04	−1.23
	T6	25.37	37.02	2.84	[-0.39, 6.06]	0.085	−12.84	−0.58	−1.49
	T7	22.77	36.84	0.42	[-2.83, 3.66]	0.802	−15.44	−0.76	−1.8
	T8	21.72	36.51	14.79	[12.96, 16.62]	<0.001^***^	−16.49	−1.09	−1.89
	Follow-up	22.07	36.56	-	-	-	−16.14	−1.04	−1.85
Resilience	T0	44.76	46.06	−16.55	[-20.17,−12.93]	<0.001^***^	0	0	−0.14
	T1	45.57	47.26	−16.94	[-20.56,−13.32]	<0.001^***^	0.81	1.2	−0.19
	T2	48.25	46.01	−13.01	[-16.75,−9.27]	<0.001^***^	3.49	−0.05	0.25
	T3	51.7	45.73	−9.28	[-13.06,−5.51]	<0.001^***^	6.94	−0.33	0.66
	T4	55.41	48.3	−8.13	[-12.07,−4.2]	<0.001^***^	10.65	2.24	0.78
	T5	55.92	46.76	−6.1	[-10.14,−2.06]	0.003^**^	11.16	0.7	1
	T6	60.8	46.05	−0.5	[-4.6, 3.6]	0.810	16.04	−0.01	1.62
	T7	62.35	47.08	0.02	[-4.11, 4.15]	0.993	17.59	1.02	1.68
	T8	64.65	46.23	−18.42	[-20.94,−15.9]	<0.001^***^	19.89	0.17	2.02
		Follow-up	62.28	47.03	-	-	-	17.52	0.97	1.67

*For Week 8, B, 95% CI, and p are from post-hoc pairwise comparisons (CG – EG) using estimated marginal means and standard errors from the linear mixed model*. ^*^, ^**^, and ^***^ indicate statistical significance at *p* < 0.05, *p* < 0.01, and *p* < 0.001, respectively.

At the prespecified primary endpoint (Week 8), the ITT analysis revealed statistically robust between-group differences: burnout decreased by 11.84 points in the Mindfulness group vs. 2.23 in controls; stress decreased by 16.49 vs. 1.09; and resilience increased by 19.89 vs. 0.17. These findings suggest that participants in the mindfulness group experienced greater reductions in stress and burnout, and greater increases in resilience, compared to controls.

According to Cohen's conventions, effect sizes of |*d*| ≥ 0.8 are considered large. In the present study, between-group standardized effects at Week 8 ranged from |*d*| = 1.2 to 2.0, corresponding to large-to-very-large magnitudes. Negative d values indicate reductions in burnout and stress, whereas positive d values reflect increases in resilience, consistent with the predefined directionality of each outcome.

Complementary analyses based on completers (*n* = 82) yielded consistent patterns in direction and magnitude, supporting the robustness of the ITT findings and serving as sensitivity analyses.

These results demonstrate robust associations between participation in the mindfulness program and favorable trajectories of burnout, stress, and resilience over time, though causal interpretations should be made with caution, given the absence of longitudinal measurement invariance testing.

As shown in Table 9, at the primary endpoint of Week 8, the mindfulness intervention produced large and highly significant effects, with the Mindfulness group showing a decrease of 11.84 points in academic burnout compared to 2.23 points in the control group, a decrease of 16.49 points in academic stress compared to 1.09 points in controls, and an increase of 19.89 points in psychological resilience compared to 0.17 points in controls. These differences were statistically robust, with effect sizes indicating strong and meaningful changes. At follow-up, the between-group differences remained substantial, with reductions in burnout of 11.42 vs. 1.92, decreases in stress of 16.14 vs. 1.04, and increases in resilience of 17.52 vs. 0.97, demonstrating that the intervention effects were sustained and clinically meaningful.

## 4 Analysis and discussion

### 4.1 Effects of mindfulness on academic stress and psychological symptoms

The findings of this study revealed that academic stress among students in the experimental group significantly decreased after the 8 week mindfulness training. Specifically, the average academic stress score dropped from 38.58 at baseline (T0) to 21.72 at the end of the intervention (T8) and remained at a relatively low level during the follow-up phase, indicating a sustained intervention effect. The between-group comparison at Week 8 and at follow-up showed a significant difference favoring the mindfulness group (*B* = 1.80, *p* < 0.001), confirming the primary intervention effect and its maintenance over time. The LMMs analysis showed a significant time × group interaction effect, suggesting that the reduction in academic stress in the experimental group was significantly greater than that in the control group.

In addition, academic burnout in the experimental group showed a significant overall decline, with particularly notable improvements in the emotional exhaustion dimension. Psychological resilience also increased significantly in the experimental group, whereas no significant changes were observed in the control group. These results demonstrate that mindfulness training has a significant effect in alleviating academic stress, reducing academic burnout, and enhancing psychological resilience, supporting H1, H2, and H3 hypotheses.

These findings align with prior research demonstrating the efficacy of MBIs in reducing academic stress among university students. For instance, a meta-analysis by Dawson et al. (2020) found that mindfulness interventions significantly reduced stress (SMD = −0.43) across 25 RCTs, particularly in high-pressure academic contexts. Similarly, a longitudinal reduction in stress among Chinese university students, with a 30-55% decrease in depressive symptoms during exam periods, is consistent with our observed 43.7% stress reduction. However, our sustained follow-up effects contrast with Galante et al. (2023), who noted a partial stress rebound in UK students post-intervention, possibly due to cultural differences in academic pressure, such as China's exam-centric system amplifying sustained benefits. These cross-study comparisons provide empirical support that the magnitude of improvement in our sample is consistent with or slightly exceeds prior mindfulness RCT conducted in comparable educational settings.

These results suggest that mindfulness training's stress reduction may be particularly robust in collectivist cultures with high academic expectations, enhancing its theoretical relevance for culturally tailored interventions. From a mechanistic perspective, mindfulness training may reduce repetitive processing of negative automatic thoughts and enhance non-judgmental acceptance of stressful events, thereby decreasing subjective stress perception.

To understand how these mechanisms operate, it is critical to distinguish between the temporary effects of mindfulness practice and its long-term dispositional impact. State mindfulness refers to temporary, context-specific mindful awareness cultivated during practice, while trait mindfulness reflects a stable, dispositional tendency to engage mindfully in daily life. The mindful state, as cultivated in our 8 week intervention, is associated with increased cognitive flexibility and psychological resilience, enabling students to maintain more effective emotional regulation and adaptive coping when facing academic stress.

A prior study supports this mechanism that brief mindfulness training can reduce stress and enhance state mindfulness, thereby contributing to more adaptive emotional regulation among university students (Sousa et al., 2021). Additionally, trait mindfulness may play a mediating role in stress reduction, as evidenced by recent research showing that trait mindfulness is associated with lower levels of perceived stress and can mediate the relationship between effort-reward imbalance and stress in university populations (Ocera et al., 2024). However, it should be noted that no formal mediation analysis was performed in the current study; therefore, any mechanistic interpretation remains tentative and exploratory rather than causal.

These findings suggest that both state and trait mindfulness contribute to stress alleviation, with trait mindfulness fostering long-term adaptive coping through sustained self-regulation. Future research could use mediation analyses to disentangle the relative contributions of state vs. trait mindfulness in academic stress reduction.

**Figure 2:**
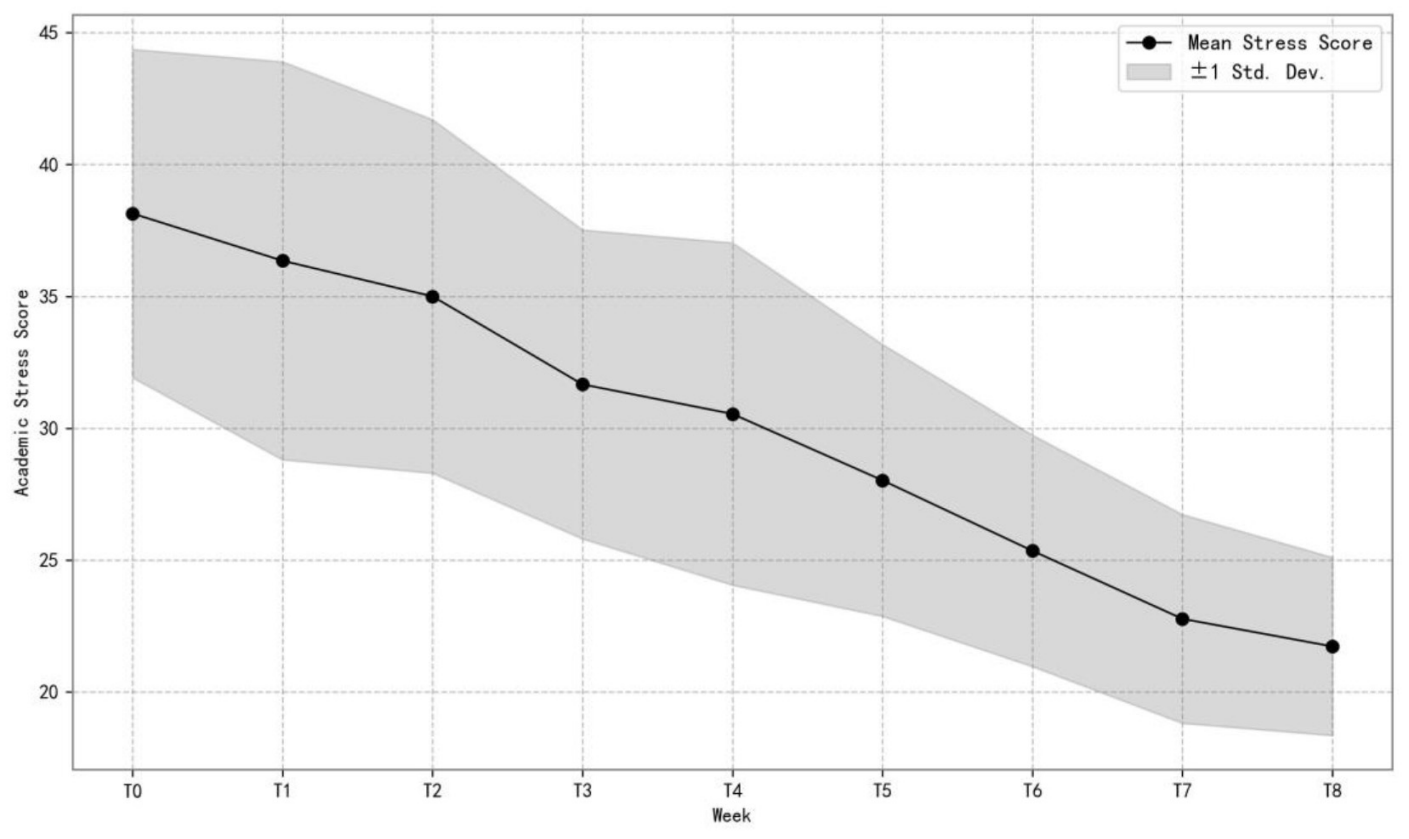
Progression of mean and SD of academic stress in the experimental group (*n* = 77).

Figure 2 illustrates the changes in the mean and standard deviation of academic stress scores in the Mindfulness Experimental Group across different time points. As shown, academic stress levels demonstrated a clear downward trajectory over time. Starting at approximately 40 points at the baseline (T0), the mean gradually decreased and reached around 22 points by T8. Meanwhile, the standard deviation around the mean also became narrower over time, indicating reduced variability in stress levels among students. Overall, students in the Mindfulness Experimental Group exhibited a sustained and relatively consistent decline in academic stress throughout the intervention period. For a detailed view of the weekly distributional changes in academic stress, see Supplementary Figure 1.

### 4.2 Trends and regulatory effects of academic burnout dimensions

This study found that 8 weeks of mindfulness training led to significant improvements in overall academic burnout. Further examination of the three dimensions revealed the following patterns: EE decreased from an average of 13.37 at baseline (T0) to 5.37 at follow-up, representing a 59.8% reduction, with the paired *t* indicating a significant effect (*t* = 15.583, *p* < 0.001). CY decreased from 13.37 to 7.00, a 47.7% reduction, also significant (*t* = 12.625, *p* < 0.001); AE increased from 13.40 to 15.33, a 14.4% improvement, with a significant paired *t*-test result (*t* = 3.489, *p* = 0.0012).

Detailed results are presented in Table 10.

**Table 10:** Changes in academic burnout dimensions from t0 to follow-up: full sample vs. completer.

**Sample**	**Dimension**	**T0 Mean ±SD**	**Follow-up Mean ±SD**	**Change (%)**	***t***	***p***
Full Sample (*n* = 153)	EE	13.83 ± 2.93	6.85 ± 4.45	−50.4	13.073	<0.001^***^
	CY	14.01 ± 3.65	7.75 ± 4.41	−44.7	11.547	<0.001^***^
	AE	13.92 ± 3.40	14.21 ± 4.09	0.10	−1.798	0.074
Completer (*n* = 43)	EE	13.37 ± 3.31	5.37 ± 0.49	−59.8	15.583	<0.001^***^
	CY	13.37 ± 3.31	7.00 ± 0.12	−47.7	12.625	<0.001^***^
	AE	13.40 ± 3.60	15.33 ± 0.47	14.40	−3.489	0.001^**^

^**^, and ^***^ indicate statistical significance at *p* < 0.01, and *p* < 0.001, respectively.

The above results indicate that mindfulness intervention not only significantly reduced the negative dimensions of academic burnout (EE and CY) but also enhanced the positive dimension (AE), demonstrating a comprehensive effect on improving students' overall academic burnout levels.

These results are consistent with prior studies on mindfulness and burnout. Cai et al. (2025) reported that mindfulness was significantly and negatively associated with learning burnout among Chinese university students, with regulatory emotional self-efficacy partially mediating this relationship. This aligns with our observed 59.8% EE reduction. However, our stronger effect on EE compared to CY (47.7%) differs from some studies, where cynicism reductions were comparable to EE (SMD = −0.45). This discrepancy may reflect contextual differences in academic stress dynamics in China, where longitudinal studies have highlighted complex interactions between academic stress and various psychological and behavioral factors Han et al. (2024). The modest AE improvement (14.4%) echoes Mulati and Purwandari (2022), who noted that resilience-enhancing interventions often yield smaller efficacy gains due to slower cognitive restructuring. Theoretically, these findings underscore mindfulness's role in modulating self-regulatory processes, particularly in high-pressure academic contexts.

From a theoretical perspective, EE and AE are primarily related to individuals' self-awareness, emotion regulation abilities, and academic goal motivation, which are core mechanisms of mindfulness training. CY reflects students' alienation and confusion regarding academic and interpersonal relationships. Its significant reduction suggests that mindfulness training is also effective in modulating interpersonal attitudes and psychological adaptation, though the extent of change is somewhat smaller compared to EE. Interview feedback further supports this interpretation: participants reported more noticeable improvements in emotional regulation and self-efficacy, while their perceived reduction in interpersonal detachment was relatively less pronounced.

Further analyses revealed that academic burnout may play a moderating role in the relationship between mindfulness training and the reduction of academic stress. Participants with lower levels of burnout showed greater reductions in stress after the intervention, whereas those with higher burnout levels also experienced stress relief but to a lesser extent. Further analyses indicated that academic burnout moderated the effect of mindfulness training on stress reduction. Students with lower burnout showed greater improvement, while those with higher burnout benefited less. This pattern is consistent with Wang et al. (2025), who found that higher academic stress predicted more severe emotional problems over time, suggesting that sustained academic strain may weaken intervention effects and require tailored motivational strategies. This finding suggests that future interventions could incorporate additional motivational reconstruction and emotional support modules for high-burnout groups to enhance the specificity and overall effectiveness of the training.

**Figure 3:**
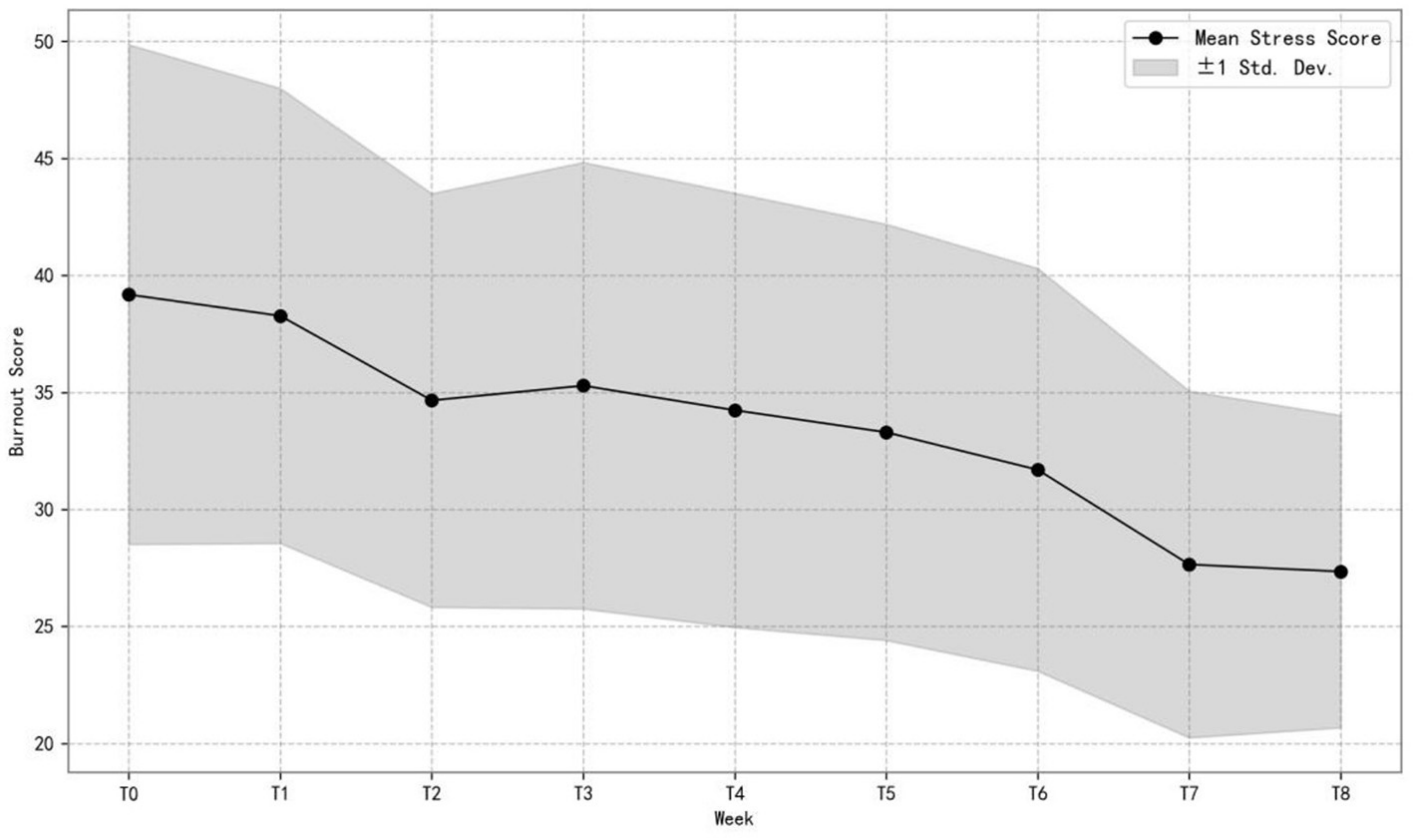
Progression of mean and SD of academic burnout in the experimental group (*n* = 77).

Figure 3 presents the changes in the mean and standard deviation of academic burnout scores in the Mindfulness Experimental Group across different weeks. We can see from the figure that the mean academic burnout scores exhibited a declining trend, starting from approximately 40 at T0. Although there was a slight fluctuation between weeks T2 and T3, scores continued to decrease thereafter, reaching around 27 by T8. Meanwhile, the standard deviation around the mean became narrower over time, indicating a reduction in variability among students. This pattern indicates that the 8 week mindfulness program effectively reduced overall burnout levels and made changes in students' behavior more consistent, verifying the H2 hypothesis. For detailed distributional information, see Supplementary Figure 2.

### 4.3 The role and significance of psychological resilience in alleviating academic stress

The findings of this study indicate that psychological resilience significantly improved following the mindfulness intervention, and this improvement was maintained throughout the 8 week intervention and the follow-up period (*p* < 0.001). Students in the Mindfulness Experimental Group demonstrated enhanced emotional regulation and psychological adaptability during the intervention, enabling them to recover more quickly from academic stressors. This suggests that psychological resilience may play a mediating or moderating role in the relationship between mindfulness training and the alleviation of academic stress.

From a theoretical perspective, previous studies have shown that psychological resilience is negatively associated with academic stress and may function as a mediating mechanism influencing the effects of mindfulness interventions (Mammarella et al., 2018; Zahra and Riaz, 2017).

A meta-analysis by Mulati and Purwandari (2022) confirmed a strong negative correlation between resilience and academic stress (*r* = −0.503), supporting our findings that resilience enhancement mediates stress reduction. However, our sustained resilience gains contrast with Western studies, such as Galante et al. (2023), where resilience effects diminished post-intervention, possibly due to lower baseline stress in individualistic cultures. In contrast, Han et al. (2024) found that Chinese students' resilience improvements were more durable in exam-centric contexts, likely due to cultural emphasis on perseverance. These cross-cultural differences highlight the theoretical importance of resilience as a dynamic buffer in high-pressure academic settings.

The current results are consistent with this view, indicating that mindfulness training may alleviate academic stress indirectly by enhancing emotional regulation, cognitive reappraisal, and perseverance toward academic goals. Future research could further test this hypothesis using correlation and mediation analyses. From a practical perspective, these findings highlight the importance of integrating psychological resilience training with mindfulness interventions in university mental health education. Cheng and Lin (2023) found that academic stress predicted burnout, which subsequently increased internet addiction, together explaining reduced wellbeing among Chinese adolescents. This highlights the importance of integrating resilience training into mindfulness interventions to strengthen students' psychological defenses and enhance long-term stress coping capacities. Strengthening students' resilience can help them build stable psychological defense mechanisms and improve their long-term ability to cope with academic stress.

**Figure 4:**
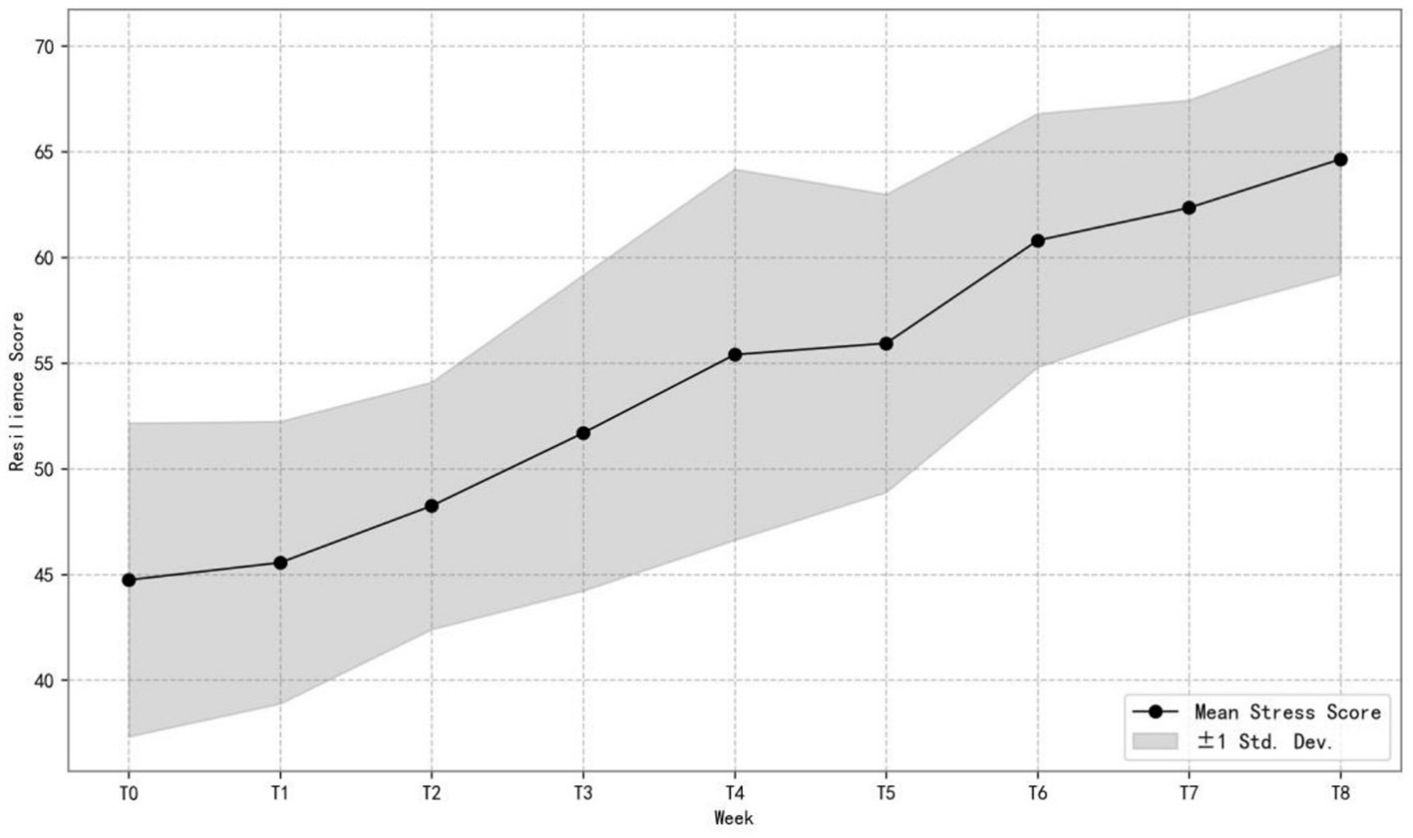
Progression of mean and SD of psychological resilience in the experimental group (*n* = 77).

Figure 4 illustrates the mean and standard deviation of psychological resilience scores for the Mindfulness Experimental Group across different weeks. We can see from the figure that the mean resilience score showed a steady upward trend, increasing from approximately 45 at T0 to nearly 65 by T8. Although the standard deviation band around the mean remained relatively wide, it expanded steadily over time, indicating continuous enhancement of psychological resilience without abnormal fluctuations in variability among students. This pattern suggests that the 8 week mindfulness program had a positive and sustained impact on students' psychological resilience. The enhancement of psychological resilience indicates that mindfulness training can strengthen students‘ coping abilities, supporting Hypothesis H3. For additional distributional details, see Supplementary Figure 3.

### 4.4 Special phenomena observed during the study and possible mechanisms

During the intervention and follow-up phases, several noteworthy phenomena were observed. First, for some participants, the reduction in academic stress was not significant during the initial stage of mindfulness training (T1). This may be attributed to the “awareness enhancement effect,” whereby individuals' attention shifts more toward internal experiences in the early stages of practice, leading to heightened sensitivity to their own tension and discomfort. As the training progressed, stress levels began to decline significantly from Week 2 onward and remained low at the end of the intervention (T8) and throughout the follow-up period.

Second, a small subset of students with high academic burnout showed low adherence to the intervention, often avoiding mindfulness practice. This may be related to motivational deficits associated with burnout, suggesting that interventions for this population should incorporate more engaging or stepwise psychological activities to lower the entry barrier and enhance participation.

Follow-up data after the intervention indicated a slight increase in stress levels among some students, although their psychological resilience remained high. This suggests that while enhanced resilience provides a buffering effect, long-term stability still requires ongoing emotional regulation and coping strategies.

Generally, these phenomena may involve three underlying psychological mechanisms. First, the awareness enhancement effect at the early stage of training may trigger emotional exposure, causing individuals to temporarily experience heightened tension or discomfort. This is consistent with Dawson et al. (2020), who noted temporary stress increases in early mindfulness training due to heightened self-awareness, particularly in high-stress cohorts. Second, motivational deficits in students with high levels of burnout may result in lower adherence to the mindfulness practice. Wang et al. (2025) similarly reported that high burnout in Chinese adolescents reduced intervention engagement by 15%, suggesting culturally tailored motivational strategies. Third, after the intervention concludes, the dynamic balance between external academic pressures and the recovery of psychological resources may account for the slight rebound in stress levels observed during the follow-up period. This aligns with Cheng and Lin (2023), who found a 10-15% stress rebound in Chinese students post-intervention, attributed to persistent exam-related pressures in collectivist cultures. These observations further support Hypotheses H1-H3, indicating that mindfulness training can effectively mitigate academic stress, ameliorate academic burnout, and enhance psychological resilience, although individual differences and early-stage adaptation effects may lead to short-term fluctuations.

**Figure 5:**
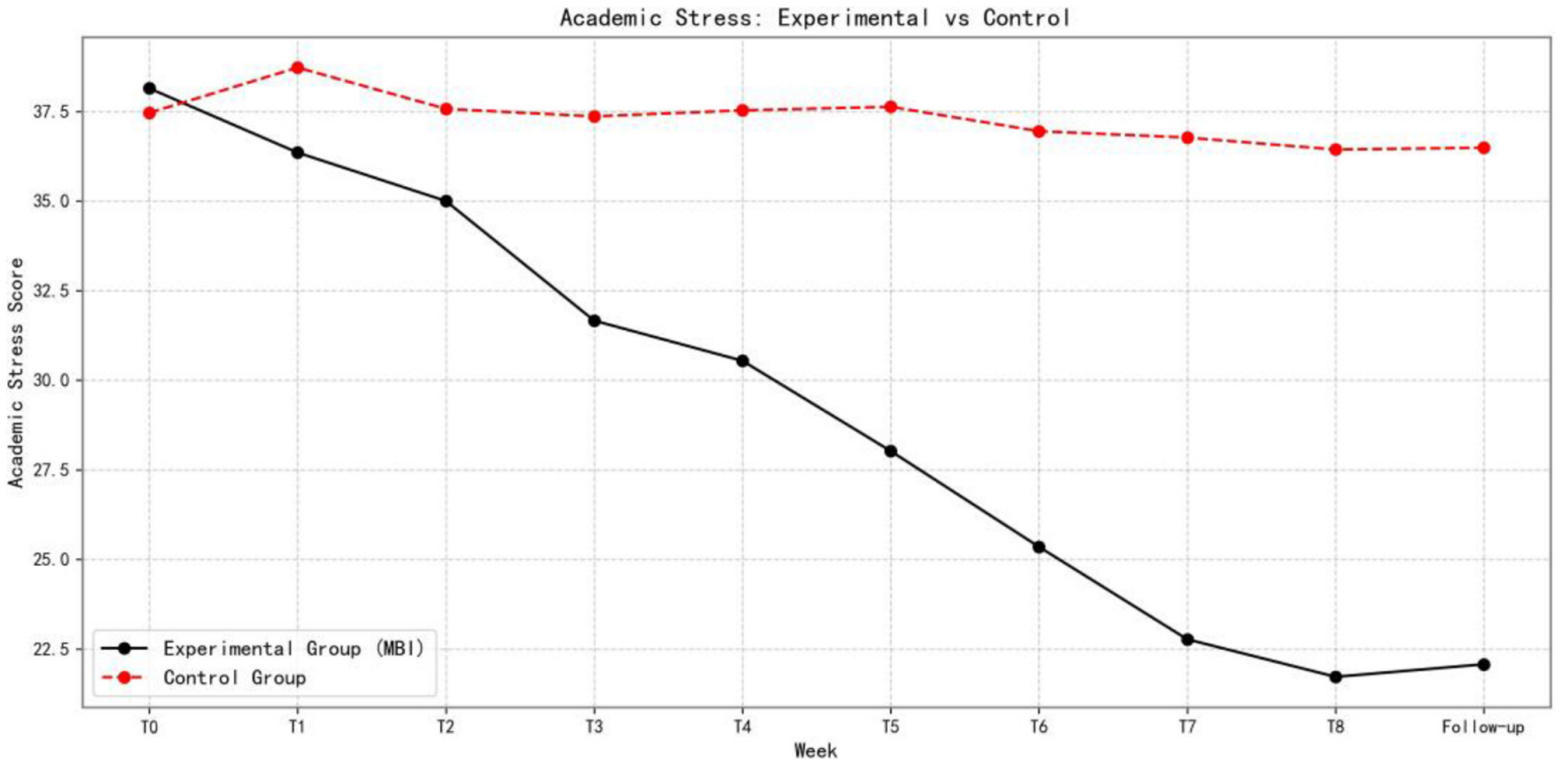
Comparison of academic stress between experimental and control groups over time (*n* = 153).

**Figure 6:**
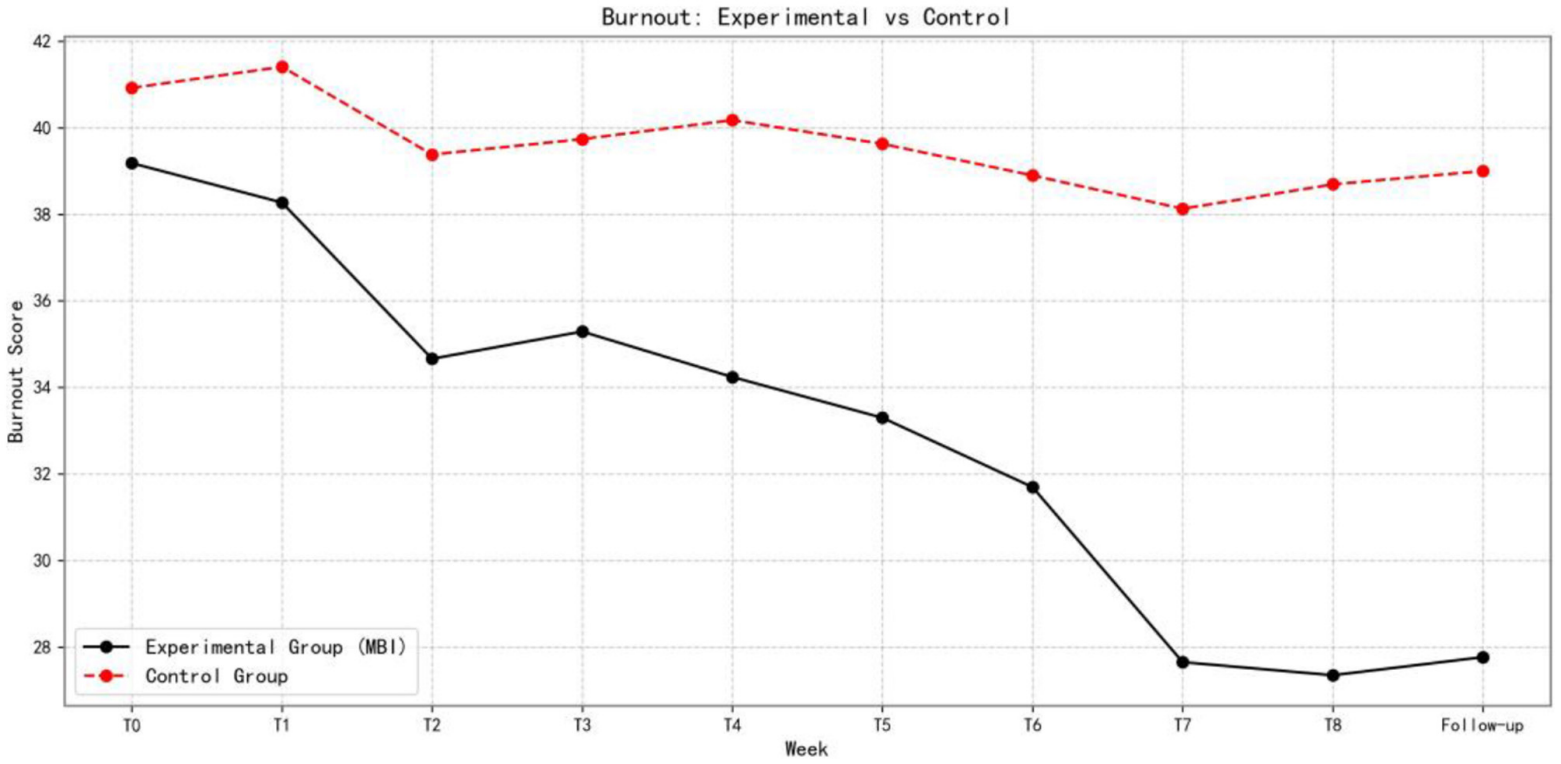
Comparison of Academic Burnout Between Experimental and Control Groups Over Time (*n* = 153).

**Figure 7:**
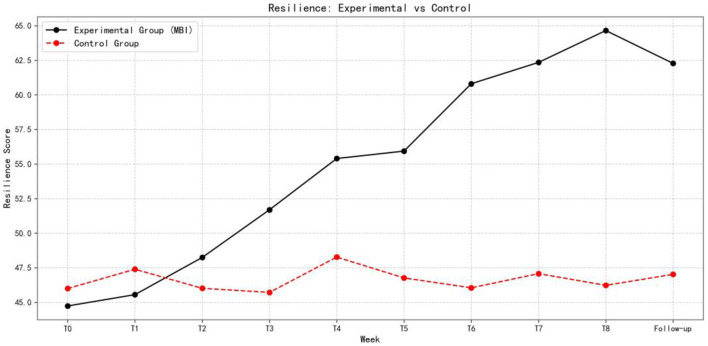
Comparison of psychological resilience between experimental and control groups over time (*n* = 153).

Figures 5–7 show the changes in academic stress, academic burnout, and psychological resilience, respectively, in the experimental and control groups over the eight-week intervention and follow-up period.

Table 11 presents the results of LMM examining the effects of the mindfulness intervention on academic burnout, psychological resilience, and academic stress in the experimental and control groups (*n* = 82). All models included participant ID as a random intercept and controlled for gender and age. *B* represents unstandardized fixed-effect coefficients, β indicates standardized effect sizes, and [95% CI] denotes the 95% confidence intervals. The *p*-values reflect statistical significance (*p* < 0.05, ^*^p < 0.01, ^**^p < 0.001). Random Intercept SD represents the estimated standard deviation of the random intercept across individuals. All models assumed a Gaussian distribution with an identity link and were used to evaluate the main effects of group and time, as well as their interaction, on each psychological outcome. The random intercept for resilience was near zero, indicating highly consistent individual trajectories in response to the intervention.

**Table 11:** Effects of mindfulness intervention on academic stress, burnout, and resilience in Full Sample (*n* = 153).

**Outcome**	**EG Mean (T0)**	**EG Mean (T8)**	**ΔEG (T8–T0)**	**CG Mean (T0)**	**CG Mean (T8)**	**ΔCG (T8–T0)**	**Group × Time B**	**95% CI**	***p***	**Cohen's *d***
Burnout	39.19	27.35	−11.84	40.89	38.66	−2.23	11.31	[8.62, 14.0]	<0.001 ^***^	−1.26
Stress	38.21	21.72	−16.49	37.6	36.51	−1.09	14.79	[12.96, 16.62]	<0.001 ^***^	−1.89
Resilience	44.76	64.65	19.89	46.06	46.23	0.17	−18.42	[-20.94,−15.9]	<0.001 ^***^	2.02

EG, Experimental Group; CG, Control Group; Δ = within-group change (T8–T0); ^***^indicates statistical significance at *p* < 0.001.

### 4.5 Study limitations and future research directions

This study achieved positive findings regarding the effects of mindfulness practice on academic burnout, psychological resilience, and academic stress, but several limitations should be noted.

First, this was not a preregistered trial, and the absence of preregistration may limit transparency and replication potential. However, full ethical approval was obtained from the Human Research Ethics Committee of the School of Education, Baoji University of Arts and Sciences (Approval No. BJWLXY-EDU-2024-012, granted April 12, 2024), and all procedures adhered to the Declaration of Helsinki. Second, the sample was drawn primarily from universities in a single region, which limits the external validity and generalizability of the findings. Third, the data relied mainly on self-reported questionnaires, which may be influenced by social desirability and subjective bias.

In addition, the study was not blinded, and the use of active control condition of psychoeducational content could have introduced expectancy or contamination effects that may partially account for group differences. Although the control condition provided weekly psychoeducational materials to maintain engagement, it functioned only as a minimal active control without mindfulness content, which may have produced limited expectancy effects. Furthermore, although intervention fidelity was monitored through attendance records (Table 1), daily homework compliance was not systematically collected due to the voluntary nature of the program; future studies should include objective logs or digital tracking to strengthen manipulation checks.

Although the Chinese versions of the instruments underwent translation, back-translation, and pilot testing, formal testing of structural validity and longitudinal measurement invariance was not conducted. In particular, for the shortened 15-item Academic Stress Scale adapted from the original 35-item version, confirmatory factor analysis and invariance testing were not performed to establish its factorial stability over time. This limitation means that observed score changes should be interpreted as associations rather than definitive causal changes in latent constructs.

To improve future research, systematic examination of measurement invariance and factorial validity is needed to ensure interpretability across time. Details are as follows:

(1) Conduct preregistered multi-site randomized trials to enhance transparency and replicability; (2) increase the sample size and improve the precision of power estimation for longitudinal models, such as simulation-based power analysis for LMM, while expanding the sample across different geographic regions to enhance representativeness and generalizability; (3) extend the follow-up duration to evaluate long-term maintenance effects and to test mediation or moderation models using longitudinal data;(4) employ multi-method assessment, such as combining physiological measures and behavioral observations, such as objective indices like exam performance, sleep quality, or heart rate variability, to improve data objectivity and accuracy; (5) incorporate more advanced psychometric evaluations, including longitudinal measurement invariance testing and multilevel reliability indices, to strengthen the robustness and interpretability of time-varying constructs; and (6) systematically report intervention fidelity, adherence rates, and ITT analyses to ensure transparency and reproducibility.

Future trials should also emphasize strategies to minimize attrition and enhance adherence to ensure adequate statistical power and reduce potential bias in longitudinal inference. In the present study, attrition-related effects on statistical power and interpretability were explicitly acknowledged and addressed through *post-hoc* power evaluation and sensitivity analyses.

## 5 Conclusion

This study employed an RCT to systematically evaluate the effects of structured mindfulness training on academic stress, dimensions of academic burnout, and psychological resilience among university students during the examination period. The mindfulness intervention significantly reduced academic stress in the experimental group relative to the control group, with a large between-group effect (Cohen's *d* = −1.89, 95% CI [12.96, 16.62], *p* < 0.001), indicating a substantial decrease in stress levels at *post-test*.

Regarding academic burnout, emotional exhaustion was notably alleviated, and academic efficacy was significantly improved, whereas cynicism showed a smaller degree of change, suggesting differential sensitivity of burnout dimensions to the intervention. Psychological resilience also increased significantly after the intervention, and this enhancement was maintained at follow-up, though without direct evidence of mediation effects. Therefore, causal or mediational interpretations should be considered preliminary until formally tested in future analyses.

However, some degree of attrition and self-report bias may have influenced the precision of estimates, and these analytic constraints should be considered when interpreting the findings. A slight rebound in stress levels was observed in some students during follow-up, suggesting that sustained emotional regulation or supplementary interventions may help maintain the benefits.

In conclusion, this study contributes empirical evidence that structured mindfulness training can alleviate academic stress and burnout symptoms among university students in a single-site context. Generalization beyond this setting should be made cautiously until replicated through multi-site, pre-registered trials with objective outcome measures. Future research could expand the sample size, strengthen fidelity monitoring, and examine long-term effects across different populations to enhance external validity and the practical value of mindfulness-based mental health programs in higher education.

## Data Availability

The data generated and analyzed in this study involve sensitive student information and must comply with the Personal Information Protection Law of China (PIPL) as well as the ethical regulations of the university. Therefore, the data are not publicly available. Aggregated and anonymized data supporting the results are provided in Tables 1–5. Researchers interested in accessing anonymized data for academic purposes may contact the corresponding author or the Ethics Committee of the School of Education, Baoji University of Arts and Sciences, at webmaster@bjwlxy.edu.cn. Requests to access the datasets should be directed to Shanwei Chen, chenshanwei@bjwlxy.edu.cn.
