# Supplementary material for: A randomized controlled trial of mindfulness: effects on academic stress, academic burnout, and psychological resilience in university students
Source: Front Psychol. 2025 Nov 27;16:1722669. doi: 10.3389/fpsyg.2025.1722669 (PMC12696859; doi:10.3389/fpsyg.2025.1722669)

**Supplementary Figure S1. Weekly Distribution of Academic Stress Scores in the Mindfulness Intervention Group**

*This figure corresponds to Figure 3 in the original manuscript.*

The kernel density distributions of weekly academic stress scores from T0 to T8 are displayed. At the early time points, the density peak was concentrated in the lower score range, and the distribution was relatively clustered. As the intervention progressed, the tendency for the distribution to spread toward higher scores diminished, with scores increasingly concentrated in lower ranges. This pattern indicates that students’ stress levels shifted toward lower values over time, reflecting an overall improvement in stress status and a decline in the occurrence of high stress scores as the intervention unfolded.


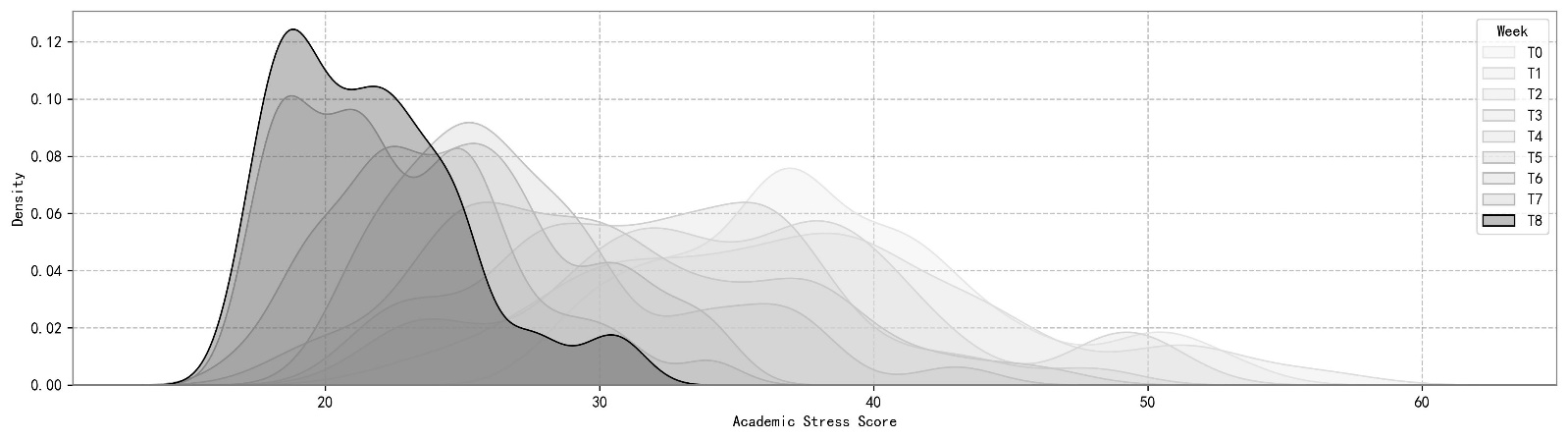


**Supplementary Figure S2. Weekly Distribution of Academic Burnout Scores in the Mindfulness Intervention Group**

*This figure corresponds to Figure 5 in the original manuscript.*

The kernel density distributions of weekly academic burnout scores from T0 to T8 are displayed. In the early weeks, the density peak was concentrated in the lower score range, with a relatively clustered distribution. As the intervention progressed, the tendency for the distribution to spread toward higher scores diminished, with more scores concentrating in lower ranges. This shift reflects a general downward movement in academic burnout scores over time, indicating the positive effect of mindfulness training in reducing burnout and a decreasing prevalence of high burnout scores among students.


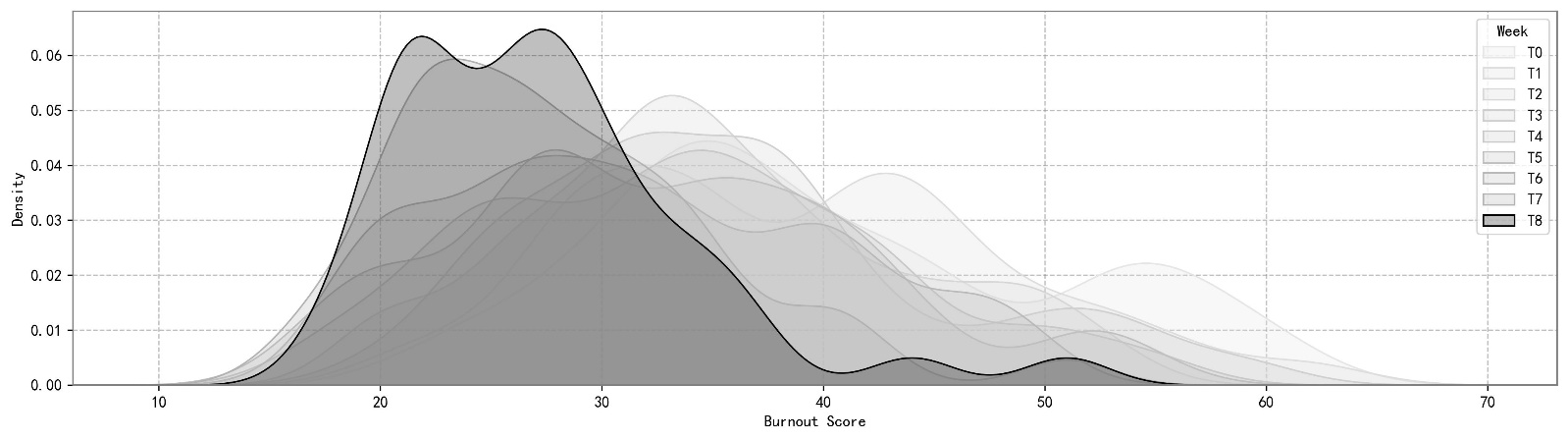


**Supplementary Figure S3. Weekly Distribution of Psychological Resilience Scores in the Mindfulness Intervention Group**

*This figure corresponds to Figure 7 in the original manuscript.*

The kernel density distributions of weekly psychological resilience scores from T0 to T8 are displayed. In the early weeks, the density peak was concentrated in the lower score range. As the intervention progressed, the distribution shifted toward higher score ranges, with a more pronounced peak, reflecting an overall upward shift in resilience scores. This pattern demonstrates the effectiveness of mindfulness training in enhancing psychological resilience, with an increasing proportion of students achieving higher resilience levels over time.


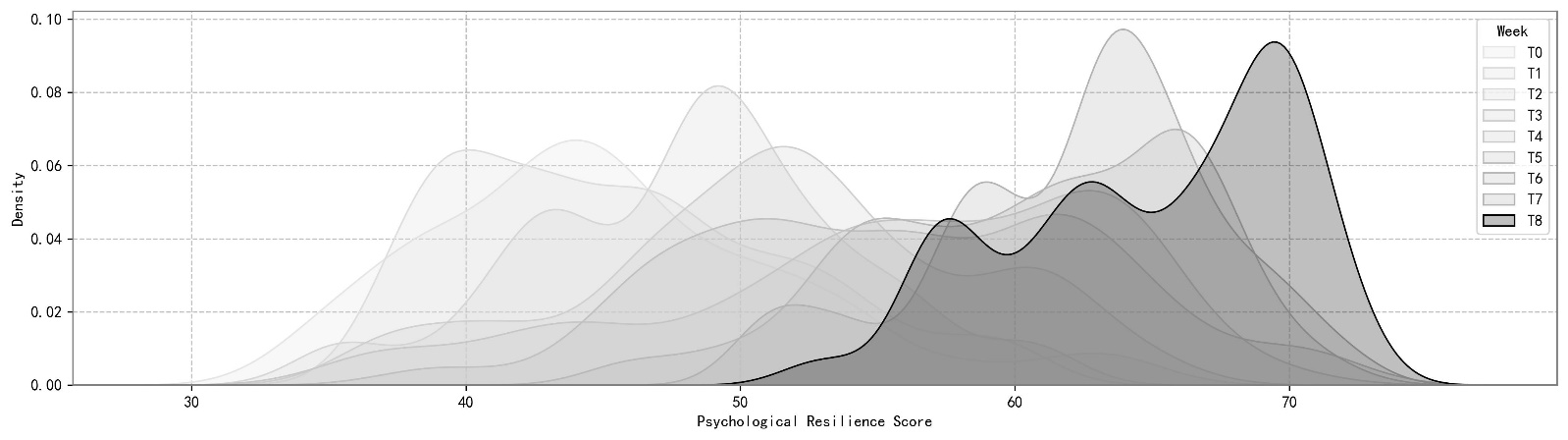

Supplement: Supplementary file 1 [file Supplementary_file_1.docx]
